# Detection of antibodies against the huntingtin protein in human plasma

**DOI:** 10.1007/s00018-023-04687-x

**Published:** 2023-01-18

**Authors:** Hélèna L. Denis, Melanie Alpaugh, Claudia P. Alvarez, Alexis Fenyi, Roger A. Barker, Sylvain Chouinard, Cheryl H. Arrowsmith, Ronald Melki, Richard Labib, Rachel J. Harding, Francesca Cicchetti

**Affiliations:** 1 Centre de Recherche du CHU de Québec, Axe Neurosciences, T2-07, 2705, Boulevard Laurier, Québec, QC G1V 4G2 Canada; 2 Département de Psychiatrie and Neurosciences, Université Laval, Québec, QC Canada; 3 Structural Genomics Consortium, University of Toronto, MaRS Building Suite 700, 101 College Street, Toronto, ON M5G1L7 Canada; 4 Laboratory of Neurodegenerative Diseases, Institut François Jacob, MIRCen, CEA, CNRS, Fontenay-Aux-Roses, France; 5 John van Geest Center for Brain Repair, University of Cambridge, Cambridge, UK; 6 Centre Hospitalier Universitaire de Montréal-Hôtel Dieu, Movement Disorders Unit, CHUM, Montréal, QC Canada; 7 Department of Medical Biophysics, University of Toronto and Princess Margaret Cancer Centre, Toronto, ON Canada; 8 Department of Mathematical and Industrial Engineering, Polytechnique Montréal, Montréal, QC Canada; 9 Department of Pharmacology and Toxicology, University of Toronto, Toronto, ON Canada

**Keywords:** Antibodies, Huntingtin protein, Plasma, Human samples, Huntington’s disease

## Abstract

Huntington’s disease (HD) is a dominantly inherited neurodegenerative disorder resulting from a CAG expansion in the huntingtin (HTT) gene, which leads to the production and accumulation of mutant huntingtin (mHTT). While primarily considered a disorder of the central nervous system, multiple changes have been described to occur throughout the body, including activation of the immune system. In other neurodegenerative disorders, activation of the immune system has been shown to include the production of antibodies against disease-associated pathological proteins. However, the existence of mHTT-targeted antibodies has never been reported. In this study, we assessed the presence and titer of antibodies recognizing HTT/mHTT in patients with HD (*n* = 66) and age- and gender-matched healthy controls (*n* = 66) using a combination of Western blotting and ELISA. Together, these analyses revealed that antibodies capable of recognizing HTT/mHTT were detectable in the plasma samples of all participants, including healthy controls. When antibody levels were monitored at different disease stages, it was observed that antibodies against full-length mHTT were highest in patients with severe disease while antibodies against HTTExon1 were elevated in patients with mild disease. Combined, these results suggest that antibodies detecting different forms of mHTT peak at different disease stages.

**Supplementary Information:**

The online version contains supplementary material available at 10.1007/s00018-023-04687-x.

## Background

Huntington’s disease (HD) is an autosomal dominant neurodegenerative disorder that is defined by a pathological CAG expansion exceeding 35 repeats in Exon1 of the huntingtin (HTT) gene. This leads to the production of a protein containing an expanded polyglutamine (polyQ) stretch referred to as mutant huntingtin (mHTT) [15, 27]. mHTT can be found in the body as a full-length protein or an Exon1 fragment [42]. While the exact contribution of different mHTT forms to the pathology is unclear, it is known that over time, the presence of mHTT within cells causes dysfunction and death, all of which leads to the clinical expression and development of overt HD features. While mHTT is classically described to drive pathology from within the central nervous system (CNS), it is expressed in all cell types and can be found in the cerebrospinal fluid (CSF), the plasma as well as within the extracellular space/matrix [12, 28, 33, 50].

Several systemic abnormalities have been described in HD, including early changes within the immune system [5]. In the CNS, this is observed by a prominent glial response [37] while in the periphery, increases in circulating levels of pro-inflammatory cytokines and chemokines associated with monocyte and macrophage alterations are measurable [8, 41, 49]. The presence of mHTT within peripheral monocytes and T lymphocytes has been suggested to correlate with disease stage and progression of caudate atrophy in patients [48]. Further evidence of interactions between mHTT and the immune system comes from a recent study which demonstrated that prolonged injection of either synthetic mHTT or HTTExon1 fibrils (HD-relevant assemblies of human HTTExon1 fragment protein, aa. 1–90) into the tail vein of mice resulted in the production of antibodies against the injected form of HTT [34]. The generated antibody response was robust, particularly after inoculation of mHTTExon1 fibrils. These findings indicate that the immune system can recognize mHTT as immunogenic and mount an antibody-targeted response against it, at least in mice.

Such antibodies—generally termed autoantibodies—are produced when the immune system targets and reacts to the individual’s own tissues, organs or specific proteins leading to inflammation, damage and dysfunction [19]. In the context of neurodegenerative diseases, there is compelling evidence to suggest that autoantibodies are present in both blood and CSF [17]. For example, autoantibodies directed against amyloid-beta [46], tau [3, 4, 29, 30, 39], α-synuclein [1, 7, 10, 22, 25, 51] and superoxide dismutase [9] have been detected in the CSF or plasma of patients with Alzheimer’s disease (AD), Parkinson’s disease (PD) and amyotrophic lateral sclerosis (ALS) as well as healthy controls. While such antibodies are consistently found, there is less consensus regarding how levels change during disease with some studies indicating higher levels at later stages [1, 4, 25, 39] while others report a decrease in autoantibody production as the disease progresses [3, 7, 10]. Variability is consistent with findings obtained in traditional autoimmune disorders and it is speculated that factors such as the subtype of antibodies detected, the affinity/avidity of antibodies for the substrate, the various bodily fluid used in antibody screens, the range of age or disease severity of patients included in the studies as well as the age/gender of the control participants could all account for discrepancies in results [2, 13, 20, 31, 44]. In contrast to the detrimental role of autoantibodies in classic autoimmune disorders, such as induction of myelin damage in multiple sclerosis (MS) [18, 21], autoantibodies against pathological proteins have also been speculated to contribute to their clearance [36]. This interpretation is strengthened by a correlation between reduced disease severity and higher autoantibody titer in patients with sporadic ALS [9].

Given the immune dysfunction associated with HD and the production of antibodies against mHTT after peripheral injection in wild-type mice [34], we developed a sensitive plasma screening platform including an indirect enzyme-linked immunosorbent and Western blot assays using multiple forms of HTT/mHTT to determine if an autoantibody response occurs in HD.

## Methods

### Study cohort

HD patients and age-/gender-matched healthy controls were recruited from HD clinics in Quebec City, Montreal and Cambridge. Each site received approval from the relevant ethical committee (CHU de Québec, #A13-2-1096; CHU of Montreal, #2015-5705; Cambridge Central Regional Motor Ethics Committee, REC #03/303 and #08/H0306/26; Cambridge University Hospitals Foundation Trust Research and Development department, R&D #A085170 and #A091246) and all participants provided informed written consent. In total, blood samples were collected from 66 gene carriers and 66 age- and gender-matched healthy controls (CTRL) (Table 1). The HD group included premanifest gene carriers (*n* = 18, 9 women and 9 men) and manifest patients with early disease (stage 1–2; *n* = 24, 11 women and 13 men) or advanced disease (stage 3–5; *n* = 24, 12 women and 12 men) according to clinical evaluations [5, 40]. A questionnaire related to health issues and medication was completed by the participant and/or their caregiver on the day the blood was collected. Comorbidities were obtained from medical information provided by the questionnaire (Table 1). A routine blood count was concurrently performed for each individual (Table 1) from blood retrieved in dipotassium ethylenediaminetetraacetic acid coated tubes (BD Vacutainer, Cat#367861). According to the reference range [16], no subject with abnormal blood counts (i.e., blood cell count, haematocrit, mean corpuscular volume, mean corpuscular hemoglobin, mean platelet volume) was recruited in the study. The mean pathological CAG repeat length within HD patients was 42 ± 2. Clinical evaluations, including the total score of the Unified Huntington’s Disease Rating Scale (UHDRS) [26] and total functional capacity (TFC) [26] were conducted within 6 months of blood collection, with the majority having been performed on the same day as blood sampling (Table 1).

**Table 1:**
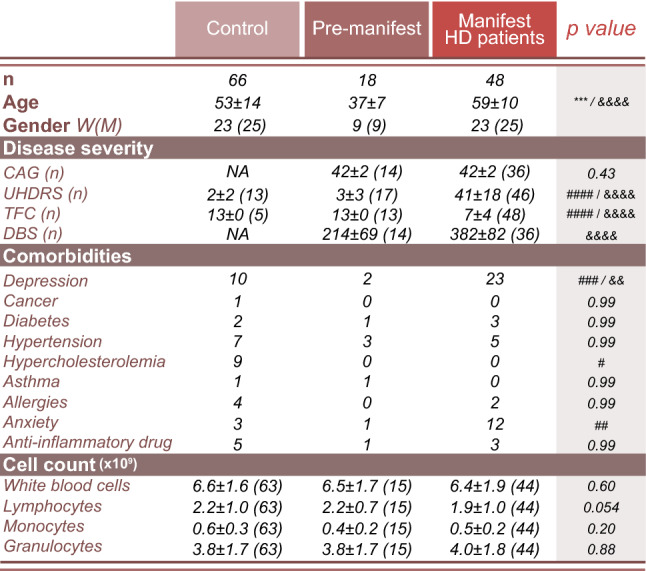
Participant information

Disease severity was evaluated within 6 months of blood sampling. Comorbidities were determined from medical information reported by the participant or caregiver. Statistics reported in the table are main effects when the model was not significant and post-tests where there was a significant difference between groups. Statistical analyses: Normality test used Agostino & Pearson test. CAG and DBS: Mann–Whitney test; Age, TFC, all comorbidities, white blood cells, lymphocytes and monocytes: Kruskal–Wallis; UHDRS and granulocytes: one-way ANOVA. test. *p* values: as indicated in table, symbols referred to the following comparisons: * control vs. pre-manifest; & pre-manifest vs. manifest HD patients; # control vs. manifest HD patients (*p < 0.05; **p < 0.01, ***p < 0.001, ****p < 0.0001)

Abbreviations: *DBS* Disease Burden Score, *W* Woman, *HD* Huntington’s disease, *M* Man, *NA* not applicable/available, *n* number, *TFC* Total Functional Capacity, *UHDRS* total score of the Unified Huntington’s Disease Rating Scale

### Human plasma isolation

Two citrate blood collection tubes (BD Vacutainer, Cat#363083) per patient were centrifuged 2 × 15 min (min) at 2500*g* at room temperature (RT). The supernatant was harvested, aliquoted and stored at − 80 °C within 2 hours (h) of sampling.

### Production of HTT proteins

All HTT protein samples (wild-type full-length HTT(Q23), full-length mHTT(Q54), Exon1-deleted HTT spanning residues 80–3144 (HTTΔExon1)) were purified, as previously described [23, 24]. Briefly, proteins were produced by baculoviral transduction in Sf9 insect cell culture and purified by FLAG-affinity chromatography and size-exclusion chromatography. pET32a-HD16Q and pET32a-HD46Q were gifts from Pamela Bjorkman (Addgene plasmid # 11487; http://n2t.net/addgene:11487; RRID: Addgene_11487 and Addgene plasmid # 11515; http://n2t.net/addgene:11515; RRID: Addgene_11515 respectively). HTTExon1(Q16) and mHTTExon1(Q46) were expressed in *E. coli* and purified by Ni-affinity and size-exclusion chromatography, as previously described [6]. The cDNA corresponding to full-length Ataxin3 with 80 glutamine repeats (RRID: Addgene_22129, a gift from Henry Paulson) was subcloned into pFBOH-SBP-TEV and verified by DNA sequencing. Ataxin3 Q80 was produced by baculoviral transduction of this construct in Sf9 insect cell culture. Cells were resuspended in 20 mM HEPES pH 7.4, 300 mM NaCl, 5% (*v*/*v*) glycerol, 1 mM TCEP supplemented with protease inhibitors and benzonase. The cell suspension was lysed by sonication and the clarified lysate was incubated with Talon resin (Cytiva). Resin was washed with a purification buffer supplemented with 5 mM imidazole and protein eluted with a purification buffer supplemented with 300 mM imidazole. Eluted protein was further purified by gel filtration using a S200 16/60 column equilibrated in the purification buffer. All samples were aliquoted and flash frozen in liquid nitrogen prior to use. Protein purity was confirmed by SDS-PAGE and sample concentration was measured using a nano drop.

### Western blot assays

#### Quantification of HTT/mHTT-reactive antibodies

Detection of denatured HTT/mHTT was assessed using Western blotting. To test this, each HTT sample (HTT/mHTT/ HTTΔExon1/HTTExon1/mHTTExon1), as well as commercial Ataxin3 (GenWay, Cat#GWB-P1694D) and Ataxin 3 Q80, were diluted in 1X Laemmli buffer and boiled at 95 °C for 5 min. Five nmol of each protein was then run on an 8% SDS-PAGE gel for 1 h 20 min at 100 V. After migration, proteins were transferred onto Millipore Immobilon-fluorescent low 0.45 μm PVDF membranes (Sigma Millipore, Cat# IPFL00005) O/N at 20 V. Membranes were removed after the transfer and total protein staining was performed using a REVERT kit (LI-COR Biotechnology, Cat#926-11011) according to the manufacturer’s instructions (Supplementary Fig. 1i). Membranes were then blocked for 45 min in 5% milk powder (Bioshop, Cat#SKI400) in phosphate buffered saline with 0.1% Tween 20 (PBST) and incubated O/N with processed plasma (dilution 1/250) from human samples (Supplementary Fig. 1k) or injected mice (Supplementary Fig. 1j) diluted in the same blocking buffer. Membranes were washed with PBST and incubated with HRP-goat anti-human Fc-gamma fragment specific secondary antibody (1/20,000, Jackson ImmunoResearch, Cat#109-035-098) or HRP-horse anti-mouse IgG (1/20,000, Cell Signaling, Cat#7076) diluted in the same blocking solution for 45 min at RT. They were then washed and HRP signal was revealed after a 2-min incubation in chemiluminescence with Immobilon Forte western HRP Substrate (Merck-Millipore, Cat#WBLUF0500) using a myECL imager (ThermoFisher, #G2236X). Band intensity was evaluated with the Odyssey Imaging System (Odyssey, Li-Cor). Human plasma IgG signals against each protein were corrected to REVERT signal intensity (Supplementary Fig. 1i) and to total IgG plasma content (Supplementary Fig. 1d–e).

#### ELISA

Antibodies against HTT, mHTT and HTTΔExon1 deletion were titered using an indirect homemade ELISA and purified plasma (Supplementary Fig. 1f). A 96-well plate (Nunc MaxiSorp™ Flat-bottom plate, Invitrogen, Cat#44-2404-21) was coated by incubation with 100 µL of 0.1 M PBS solution containing 10 µg/mL of HTT proteins of various lengths or Ataxin3 O/N at 4 °C. The plate was then washed twice with 0.1 M PBS and blocked with 5% milk powder (Bioshop, Cat#SKI400) in PBST for 2 h at RT and washed twice with 0.1 M PBS. To detect the antibody, 100 µl of purified plasma diluted in blocking solution (human: dilution factor from 80 to 2000, mice: dilution factor from 50 to 10,000) was added to the plate O/N at 4 °C. The plate was then washed 6 times with 0.1 M PBS and incubated for 45 min at RT with HRP-goat anti-human Fc-gamma fragment secondary antibody (1/4000, Jackson ImmunoResearch, Cat#109-035-098) or HRP-horse anti-mouse IgG (1/4 000, Cell Signaling, Cat#7076) diluted in blocking solution. After incubation with the secondary antibody, the plate was washed 6 times with PBST and the 3,3′,5,5′-tetramethylbenzidine (e-Bioscience, Cat# 00-4201-56) substrate was added for 8 min at RT. The reaction was stopped by addition of 0.18 M sulfuric acid and signal intensity at 450 nm was measured using a multi-detection microplate reader (Synergy HT, BioTek).

### Statistical analyses

For Table 1, comparisons between groups were obtained using Unpaired *t*-tests or Mann–Whitney tests. Differences between plasma and purified plasma were determined using Wilcoxon matched pairs signed rank test (Supplementary Fig. 1b). For analyses pertaining to Supplementary Fig. 1c, data were compared to a theoretical mean ratio of one using a One-sample *t*-test. A theoretical ratio of one was used as it corresponds to an equal concentration of IgG between plasma and purified plasma. Correlation between IgG concentration and age were assessed using simple linear regressions (Supplementary Fig. 1e).

**Fig. 1:**
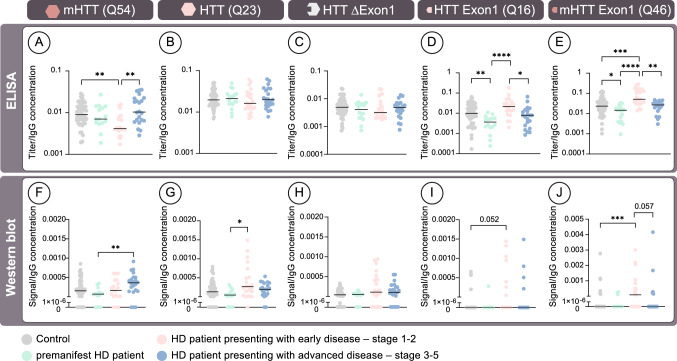
HTT and mHTT-reactive antibody response. Detection of antibodies against mHTT(Q54) (**a**, **f**), HTT(Q23) (**b**, **g**) HTT with Exon1 deletion (HTTΔExon1) (**c**, **h**), HTTExon1 (**d**, **i**), mHTTExon1 (**e**, **j**) was performed by ELISA (**a–e**) or Western blot (**f–j**) assays. Purified plasma from premanifest patients (*n* = 18) and age/gender-matched healthy controls (*n* = 18) as well as HD patients with early disease (stage 1-2, *n* = 24) or advanced clinical features (stage 3–5, *n* = 24) and age-gender matched healthy controls (*n* = 24/24) were tested. For western blot detection, signal was absent from 21 (HTT with Exon1 deletion), 100 (HTTExon1) and 93 (mHTTExon1) of the 132 participants. Statistical analyses: Kruskal–Wallis test followed by Dunn’s multiple comparisons test (*p < 0.05; **p < 0.01, ***p < 0.001, ****p < 0.0001). Abbreviations: *HTT* Huntingtin protein, *IgG* Immunoglobulin gamma, *mHTT* mutant huntingtin protein

**Fig. 2:**
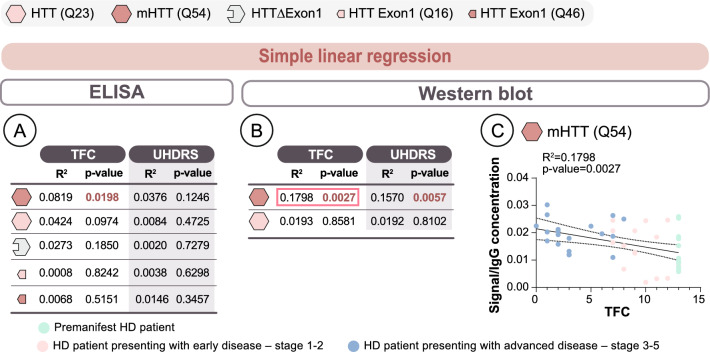
Correlations with disease features. To evaluate the relationship between the antibody response and disease metrics, we performed simple linear regression (**a–c**) for both ELISA and Western blot data. All HD gene carriers were included in the analysis. *P*-values: as indicated in the figure. Abbreviations: *HTT* Huntingtin protein, *IgG* Immunoglobulin gamma, *mHTT* mutant huntingtin protein, *TFC* Total Functional Capacity, *UHDRS* Unified Huntington’s Disease Rating Scale

Effects of the demographic variables age and sex on IgG concentration were evaluated using a Two-way ANOVA (Table 2). This model failed the test of normality. To produce both a normally distributed data set and a linear model for regression analyses, ELISA data underwent a natural logarithmic transformation and Western data a square root transformation. After the two respective data transformations, the residuals were both randomly and normally distributed for linear regression analyses and normally distributed according to a Shapiro–Wilk test for ANOVA. Therefore, all data shown in Table 2 and Fig. 2 were performed with transformed data. When the entire data set was considered, age affected several of the investigated proteins and therefore comparisons between controls and patients were performed using student’s unpaired *t*-test following a weight adjustment for age. Given the increased antibody level in mHTT and HTT as measured by Western blotting, the relationship between protein levels in HD gene carriers and CAG repeat length/DBS was evaluated using a simple linear regression. Finally, the relationship between disease stage and antibody levels in HD patients was tested using a simple linear regression. In Fig. 1, the relationship between age and antibody levels in HD patients was found to be absent, so graphs were generated using absolute values. Group differences in the antibody responses obtained by ELISA and Western blot assays were then evaluated to ensure normality of the data sets using Agostino-Pearson Omnibus k2. As unequal variances were detected between groups, a nonparametric test was used (Krustal-Wallis test). Frequency analysis of detectable antibody signal for mHTT/HTTExon1 Western blot was performed using a chi-square test. For Fig. 2, simple linear regressions were performed to evaluate potential relationships between disease metrics and antibody levels. Simple linear regressions were performed using the lm function in *R*. Unadjusted *R*^2^ values were reported. Outliers were removed for one-way ANOVA analyses but were included for all regression analyses except for HTT and mHTT Western blotting data for which they were eliminated as their inclusion resulted in a non-linear model. Outliers were defined as data points that are more than 1.5 interquartile ranges below the first quartile, or 1.5 interquartile ranges above the third quartile. All statistical analyses were completed using Prism^®^9.3.1 (GraphPad Software) or R Studio^®^1.3.1093.

**Table 2:**
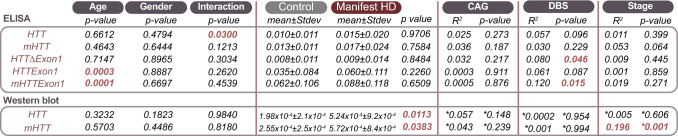
Relationship between antibodies and demographic factors

Correlation between antibody levels (ELISA) or signal (Western blot) and age/gender indicates that age influences mHTT/HTT antibody levels. Comparison of manifest HD patients and controls revealed no significant differences in antibody levels as measured by ELISA, but a significant increase by Western blot. Using results from manifest HD patients, we performed simple linear regressions to compare antibody levels/signal to CAG repeat length, DBS or stage. *Samples for which zeros were removed as there inclusion resulted in a non-linear data set even after data transformations were applied. *p* values: as indicated in table

Abbreviations: *DBS* Disease Burden Score, *HD* Huntington’s disease

^*^Outliers and zeros were removed to allow for a linear model after transformation

## Results

### Characterization of HTT/mHTT recognizing antibodies

For this study, we recruited premanifest and manifest HD gene carriers (*n* = 66) along with age/gender-matched healthy controls (*n* = 66) (Table 1) for a total of 132 participants. Both premanifest and manifest patients were selected to determine if antibodies could be detected at different stages of disease. Manifest patients displayed significantly higher clinical scores, and a higher proportion of subjects were prescribed medication for anxiety and depression, than age/gender-matched healthy controls and pre-manifest gene carriers. All other parameters were equivalent between groups aside from hypercholesterolemia, which was more prevalent in controls (Table 1). To determine if hypercholesteroleamia altered antibody levels in age/gender-matched healthy controls, the nine individuals taking medication for this condition, were compared to the remaining cohort (57 individuals) using an unpaired t-test and no significant differences were observed. Routine haematological measures were similar between HD patients and healthy controls (Table 1).

### Detection of HTT/mHTT recognizing antibodies in human plasma

Prior to commencement of the analysis of our clinical cohort, our two detection methods were optimized and validated. Details can be found in Supplementary materials. Once this validation was complete, we performed Western blot and ELISA assays on all samples. These tests revealed that antibodies were detectable in the plasma of all participants using the ELISA method and in both controls and HD gene carriers for Western blot analysis. To further explore these findings, we analyzed our complete data set to determine if demographic factors such as age and gender, which have previously been reported to impact IgG concentrations, affected the overall levels of antibodies in our experimental cohort (Table 2). A two-way ANOVA revealed that age had a significant effect on antibody levels measured by ELISA for HTTExon1 and mHTTExon1 while both age and gender affected the titer of HTT-detecting antibodies. Antibody levels detected by Western blot were not significantly affected by either factor.

After determining that antibodies are present in all participants and that age may significantly affect the titer of antibodies detected by ELISA, we next compared manifest HD patients (*n* = 48) to controls (*n* = 66) for all proteins measured by ELISA after correcting for age (Table 2). No significant differences were found. Manifest patients and age/gender matched healthy controls were compared using a student’s unpaired *t*-test after correcting for age, which indicated that antibody levels were significantly different between patients and controls for both HTT and mHTT (Table 2). Given this significant increase in manifest patients, we next explored the relationship between the detected antibodies and CAG repeat length, a critical determinant of disease as well as a predictor of age of onset (Table 2). However, no correlation between antibody levels and CAG repeat expansion were observed for any of the tested proteins using either detection method. Our previous analyses have highlighted the importance of age as a modifier of antibody levels, so we extended our analysis of CAG repeat length to include disease burden score, a metric which reflects CAG repeat length and age [38, 52]. This measure yielded significant correlations between antibodies recognizing mHTTExon1 and HTTΔExon1 (Table 2). This effect was weaker than the relationship between mHTTExon1 and age alone, and therefore does not seem to indicate that CAG repeat length is an important modifier of antibody levels.

To further explore the relationship between antibody levels and disease, we next evaluated the relationship between antibody levels and disease stage (Table 2). This analysis highlighted a modest but significant correlation between disease stage and mHTT antibody levels as measured by Western blot, as well as a trend towards a significant relationship for antibodies measured by ELISA (Table 2).

### Assessment of antibody level progression across disease

To better understand how antibody levels changed across disease progression, we performed a one-way ANOVA analysis of antibody levels in controls, pre-manifest gene carriers and manifest patients with mild or severe disease (Fig. 1). Due to the lack of relationship between age and antibody levels in HD patients, absolute values were used for this portion of the study. This analysis demonstrated a complex relationship between disease severity and antibody titers for mHTT and HTT/mHTTExon1. For mHTT, the one-way ANOVA analysis showed that pre-manifest gene carriers did not significantly differ from controls, while patients at early disease stages displayed a significant decrease in antibody levels which was lost at later disease stages (Fig. 1a). This fluctuation across disease stage was absent for the two disease unrelated proteins, namely HTT and HTTΔExon1 (Fig. 1b, c). In fact, antibodies recognizing these two proteins were found at identical levels in all groups. This contrasted with the antibody levels against the mHTT/HTTExon1 proteins which, when compared to controls, were significantly decreased in pre-manifest patients and increased in patients with early manifest disease. Oddly, manifest patients with advanced disease had antibody levels that were significantly lower than patients with early manifest disease, such that the advanced manifest group did not significantly differ from controls (Fig. 1d, e).

In contrast, we observed a progressive increase in mHTT antibodies measured by Western blotting as disease progressed, with premanifest gene carriers having significantly lower levels than patients at advanced stages of disease (Fig. 1f; Controls vs. patients with stage 3–5 disease *p* = 0.08). For antibodies detecting HTT, a slightly different pattern was observed with both manifest groups showing similar levels, although only early manifest patients reached statistical significance when compared to premanifest gene carries (Fig. 1g). Finally, no changes in antibody signal against HTTΔExon1 measured by Western blot was observed between any groups (Fig. 1h).

The rarity of participants with detectable levels of mHTT/HTTExon1 antibodies using Western blot techniques made statistical comparison difficult. However, significant increases were observed in patients with early manifest disease as compared to controls (Fig. 1i, j). This finding was supported by a frequency analysis performed using a chi-square test, which showed that individuals with detectable antibody levels were more common in the early manifest group than in the control group (*p* = 0.02).

Taken together, these results suggest that the specificity of stage 1–2 patients’ antibodies is possibly more directed to mHTTExon1 fragments than mHTT full-length. By contrast, samples from patients with advanced disease (stage 3–5) have more antibodies against the full-length mHTT.

### Assessment of the relationship between antibody levels and disease features

Combined, our findings demonstrate that all individuals express antibodies that are capable of recognizing multiple forms of HTT. Furthermore, the degree of detection of full-length HTT/mHTT antibodies is elevated in manifest HD patients and mHTT antibody levels correlate with disease stage. To better understand the relationship between antibody levels and disease features, we performed simple linear regression analyses to compare all five different protein forms to disease metrics (Fig. 2a–c). Of all the protein forms assessed by ELISA, only full-length mHTT showed a statistically significant, but very weak, association with disease symptom severity, as measured by TFC (Fig. 2a).

Correlations between antibody levels detected by Western blot and disease features yielded similar but slightly more robust findings to those of the antibodies measured by ELISA (Fig. 2b, c). Specifically, mHTT significantly correlated with both TFC and UHDRS. While significant correlations were observed, all were relatively weak, indicating that the severity of disease features only explained a small percentage of the change in antibody levels.

### Detection of antibodies against another polyQ protein

Overall, our analyses suggest that manifest HD patients, either stage 1–2 or stage 3–5 depending on the protein used to detect antibodies, have more antibodies detecting mHTTExon1 than healthy controls which could indicate a polyQ-specific autoantibody response in this population. Multiple proteins contain polyglutamine tracts, so it is therefore possible that some of the autoantibodies detecting mHTT also recognize other polyglutamine-containing proteins. We, therefore, evaluated if the increased mHTT-recognizing antibody titer present in HD patients was also present for another polyQ-containing protein, namely Ataxin3 [45]. We first identified the optimal Ataxin3 coating concentration for the ELISA plate using a commercial antibody (Supplementary Table 1). Detection of Ataxin3 with either a normal (Supplementary Fig. 2a) or expanded polyQ stretch (Supplementary Fig. 2b) was not increased in stage 3–5 HD patients, as measured by either ELISA or Western blot, which indicates that the heightened response against HTT is not primarily against the polyQ repeat. However, it should be noted that a strong trend towards an increase was observed for Ataxin3 with an expanded polyQ stretch, which could indicate that at least some of the antibodies measured in patients may bind directly to the polyQ stretch.

## Discussion

This study provides evidence that antibodies recognizing different forms of HTT/mHTT are present in human plasma. With the use of multiple forms of HTT/mHTT, we were additionally able to establish and characterize the presence of antibodies in purified human plasma using both ELISA and Western blot assays. We have identified a previously unreported aspect of HD pathology by demonstrating that there is an antibody response against HTT/mHTT in samples from 66 HD gene carriers and 66 age- and gender-matched healthy controls and that this response is amplified at certain stages of disease.

Data presented in this manuscript pertain to auto-HTT IgG antibodies. In humans, autoantibodies are of the IgM and IgG isotypes [2]. IgM autoantibodies play a housekeeping role while IgG autoantibodies are usually high-affinity, somatically mutated antibodies of which the expression reflects a pathologic process [19]. It was previously thought that most autoantibodies were IgM, but additional study has highlighted the prominence of IgG isotypes and indicated that their number is influenced by age, gender and disease [35]. In light of these findings, we focused our study on high affinity antibodies by quantifying the presence of auto-IgG antibodies in HD.

Our analyses revealed an increase in the number of antibodies present in manifest patients as compared to healthy age/gender-matched controls. While certain populations of antibodies were solely elevated in patients with stage 3–5 HD, others were additionally raised in stage 1–2 patients, indicating that increased production of mHTT recognizing antibodies is an early feature of disease which continues to change throughout the course of disease. This elevation is consistent with previous studies reporting that there is a general increase in the production and release of antibodies against autoantigens with age and disease [47], presumably as cells die and release more immunogenic antigen. The previously reported increase in inflammatory markers throughout disease progression is also likely to influence the changes in antibody levels [14]. However, the differing patterns found for the various HTT forms could indicate that antibody levels are impacted by other factors. From the current study, it is not possible to determine if the antibodies present in HD patients were initially raised against HTT, mHTT or both. However, the lack of increase in HTT-reactive antibodies (Fig. 1), as well as the absence of correlation between HTT-reactive antibody titers and clinical scores (i.e., TFC) (Fig. 2) could suggest that at least a subset of the antibodies recognizing mHTT are not the same as those detecting HTT, or that the affinity of these antibodies is stronger for mHTT than for HTT. The idea that the expanded CAG repeat is important for detection of the full-length protein is further supported by the Ataxin 3 data which showed a trend towards an increase in severe manifest patients that mirrored the results from the full-length mHTT protein (Supplementary Fig. 2). Further work with additional CAG repeat containing proteins would be required to confirm this hypothesis, especially since the length of the CAG repeat in the ataxin protein was longer than that observed for mHTT.

According to our data, the epitopes favoured by antibodies changed with disease severity and age. Specifically, our results show a transient increase in antibodies against mHTT/HTTExon1 in stage 1–2 patients which was contrasted by a steady increase in mHTT detecting antibodies measured by western blot across disease (Fig. 1). This transient detection of Exon1 is intriguing and would suggest that Exon1 fragments are more prevalent in the blood earlier in disease, with full-length forms more predominant in later disease. This hypothesis would be consistent with the idea that levels of soluble HTT decrease with disease progression and that small N-terminal fragments are more prone to aggregation than the full-length protein [32, 43]. While the presence of the alternatively spliced Exon1 fragment could explain this transient increase in early manifest HD patients, it does not explain the presence of HTT-recognizing antibodies in the plasma of healthy controls. For HTT-directed antibodies to be present, HTT release into the plasma must occur under physiological conditions. Recent work supports this idea through the demonstration that both forms of the protein are actively secreted by neurons [11]. Therefore, this process is likely to be a contributing factor to the generation of HTT autoantibodies in HD patients as well as in healthy individuals. One question that remains to be answered is how HTT/mHTT species may differ between controls and patients at various stages of disease. To date, there is no consensus regarding the type of HTT/mHTT forms present in different bodily fluids, and it is quite possible that there is a large spectrum of HTT/mHTT epitopes present in HD patients and healthy controls, including some outside of Exon1. It is additionally probable that the relative concentrations of these different HTT proteoforms would influence the HTT autoantibody titer. Therefore, further work will be needed to both characterize the HTT proteoforms measurable in plasma as well as to better delineate the epitopes recognized by circulating HTT-reactive antibodies as they arise during the disease process.

## Conclusion

Using two complementary methods, we show that HTT/mHTT detecting antibodies are present within the plasma of all tested participants, including healthy controls. We further demonstrate that their levels change in HD patients as disease progresses. Specifically, HTT/mHTTExon1 recognizing antibodies are predominant in early manifest HD followed by higher levels of mHTT antibodies later in disease. Additional work is waranted to evaluate the relationships between the levels of different forms of plasma HTT and antibody levels.

## Supplementary Information

Below is the link to the electronic supplementary material.

Supplementary file1 (PDF 941 KB)

## Data Availability

The datasets used and/or analyzed during the current study are available from the corresponding author on reasonable request.
