# Supplementary material for: Detection of antibodies against the huntingtin protein in human plasma
Source: Cell Mol Life Sci. 2023 Jan 18;80(2):45. doi: 10.1007/s00018-023-04687-x (PMC9849309; doi:10.1007/s00018-023-04687-x)
Supplement: Supplementary file 1 — Supplementary file1 (PDF 941 KB) [file 18_2023_4687_MOESM1_ESM.pdf]

Detection of antibodies against the huntingtin protein in human plasma

Hélèna L. Denis[1,2], Melanie Alpaugh[1,2], Claudia P. Alvarez[3], Alexis Fenyi[4], Roger A. Barker[5], Sylvain Chouinard[6], Cheryl H. Arrowsmith[3,7], Ronald Melki[4], Richard Labib[8], Rachel J. Harding[3*], Francesca Cicchetti[1,2*]

Correspondence should be addressed to either F.C. or R.J.H.
Francesca Cicchetti, Ph.D.
Centre de Recherche du CHU de Québec
Axe Neurosciences, T2-07
2705, Boulevard Laurier
Québec, QC, G1V 4G2, Canada
Tel #: +1 418 656 4141 ext. 48853
E-mail: francesca.cicchetti@crchudequebec.ulaval.ca

Rachel J. Harding, Ph.D.
Structural Genomics Consortium
University of Toronto
MaRS Building Suite 700
101 College Street
Toronto, ON, M5G1L7, Canada
Tel #: +1 416 946 3795
E-mail: rachel.harding@utoronto.ca

Supplementary Methods

Mouse plasma isolation

To validate our assays, mouse plasma samples from previous studies conducted in the laboratory were utilized[34]. More specifically, samples were retrieved from a study in which 8-week-old male C57BL/6NCrl mice received intravenous injections of 3.5 µg of mHTT Exon1 fibrils with 48 polyQ repeats (n=3) or BSA (n=3) as a negative control in 100 µL of isotonic saline every 2 weeks for 3 months. mHTTExon1 fibrils were produced and purified, as previously described[36]. To obtain the titer of antibodies recognizing full length HTT or mHTT (**Supplementary Fig. 1g and 1j)**, blood was collected by cardiac puncture and plasma was isolated [34].

Plasma antibody enrichment

To remove blood components smaller than 100 kDa, 100 µL of plasma and 2 mL of 0.1 M Phosphate-Buffered Saline (PBS) were added to the top of a molecular weight exclusion Amicon®Ultra-4 Centrifugal Filter Unit filter (Sigma Millipore, Cat#UFC810024) (**Supplementary Fig. 1a**). The tube was then centrifuged at 3000g for 15 min at RT. After centrifugation, any plasma that did not pass through the filter (i.e. components larger than 100 kDa) was washed again with 2 mL of 0.1 M PBS prior to centrifugation at 3000g for 15 min at RT. After the final centrifugation step, the remaining volume was adjusted to 100 µL. Total protein in plasma and purified plasma was quantified using a BCA Protein Assay Kit (Thermofisher, Cat#23225) (**Supplementary Fig. 1b**).

Western blot assays

Quantification of total plasma IgG antibodies

Total Immunoglobulin G (IgG) concentration in raw and purified plasma (**Supplementary Fig. 1c-d**) of participants was obtained by Western blot assays. Briefly, 1 µL of plasma was diluted in 49 µL of water (1/50). Two µL of the resultant solution was mixed with 18 µL of Laemmli 1X buffer and boiled at 70°C for 10 min. Samples were run on an 8% sodium dodecyl sulfate-polyacrylamide gel (SDS-PAGE) for 1 hr and 20 min at 100 V. After migration, proteins were transferred onto Amersham Hybond 0.45 µm polyvinylidene difluoride (PVDF) membranes (GE Healthcare, Cat#10600023) overnight (O/N) at 20 V. Membranes were removed after the transfer and immersed for 45 min in blocking solution (5% milk powder (Bioshop, Cat#SKI400) and 0.5% serum bovine albumin (Bioshop, Cat#ALB001) in 0.1 M PBS containing 0.1 % Tween 20 (Fisher BioReagents, Cat#BP337500) (PBST)). Membranes were then incubated with the horseradish peroxidase (HRP)-goat anti-human Fc-gamma fragment specific secondary antibody (1/20 000, Jackson ImmunoResearch, Cat#109-035-098) diluted in the same blocking solution for 45 min at RT. Membranes were washed and HRP signal was revealed after a 2-min incubation in chemiluminescence with Immobilon Forte western HRP Substrate (Merck-Millipore, Cat#WBLUF0500) using a myECL imager (ThermoFisher, #G2236X). Band intensity was evaluated with the Odyssey Imaging System (Odyssey, Li-Cor).

Supplementary Results

IgG enrichment

Several proteins within the plasma can give non-specific signal in an ELISA or Western blot assay. To eliminate false positive and false negative reactions, we enriched the IgG antibody content in plasma samples using a size-specific molecular weight cut-off filter (**Supplementary Fig. 1a**). Enriched plasma revealed a significant reduction of total protein content following processing (**Supplementary Fig. 1b**) while the total IgG ratio – the signal intensity from plasmatic

IgG/purified plasmatic IgG obtained by Western blot – remained comparable (**Supplementary Fig. 1c**). This stable concentration indicates that IgG eliminated during the enrichment protocol is minimal. After validating our method, we measured total IgG in each sample by Western blot and found a high degree of inter-subject variability (**Supplementary Fig. 1d**). Given that age[49] and anti-inflammatory medication[3] have previously been reported to affect plasma IgG content, we explored the possibility that these two factors may contribute to the variability in our data. Linear regression analysis revealed that age significantly correlated with IgG (**Supplementary Fig. 1e**). Therefore, to prevent age-related differences in total IgG content from confounding our assessment of HTT antibody levels, particularly between pre-manifest and manifest patients, all antibody levels were normalized over IgG. To assess potential effects of anti-inflammatory medication, we consulted information from the patient which was available to us and determined that 9 participants reported having taken anti-inflammatory medication (e.g. ibuprofen) 48 hours (hrs) prior to blood withdrawal. These 9 subjects were evenly balanced between groups and their IgG levels did not significantly differ from individuals who had not been prescribed such medication.

Optimization of ELISA and Western blot assays

To determine if any of the IgG antibodies recognized HTT or mHTT, we optimized both ELISA (**Supplementary Fig. 1f-h**) and Western blot (**Supplementary Fig. 1i-l**) assays. To validate our ELISA protocol (**Supplementary Fig. 1f**), we used commercial antibodies to confirm the appropriate concentration of HTT substrate and designed antibody controls which were included in all subsequent experiments. To determine the optimal HTT substrate concentration, we tested a range of concentrations (from 1 μg/mL to 20 μg/mL) in combination with two commercial antibodies: D7F7 (**Supplementary Table 1**) and EPR5526 (**Supplementary Table 1**). Saturation

binding for both antibodies was reached at 10 µg/mL for HTT and mHTT in 0.1M PBS (Kd_{D7F7} = 0.05 nM ± 0.01 (n = 4), $Kd_{EPR5526}$ = 0.17 nM ± 0.05 (n= 4)). Consequently, 10 µg/mL of substrate was used in all subsequent experiments.

After identifying an appropriate substrate concentration, we evaluated HTT-reactive antibody titers in plasma isolated from wild-type mice injected with Q48 fibrils, which were previously reported to have a high titer of circulating anti-mHTTExon1 antibodies[34]. Using our optimized ELISA conditions, we successfully detected the expected increase in mHTT-reactive antibodies in mice injected with Q48 fibrils as compared to mice injected with bovine serum albumin (BSA) (**Supplementary Fig. 1g**). In addition to confirming the validity of our assay, the detection of full-length mHTT after injection with Exon1 fibrils suggests that the resultant antibodies recognize multiple HTT/mHTT protein forms (**Supplementary Fig. 1g**). When testing human blood samples, we confirmed test-retest validity by analyzing the same plasma samples twice (**Supplementary Fig. 1h**). For this experiment, and all subsequent analyses, antibody titer (indicated with a dotted line in **Supplementary Fig. 1h**) was calculated and used as the measure of antibody concentration. Antibody titer was defined as the lowest dilution with clear positive signal (A_{450}≤0.2). Antibody controls were performed for each plasma sample and included wells without HTT coating and wells with HTT coating but without the secondary antibody (background curve in **Supplementary Fig. 1h**, average of A_{450}=0.05). The first measure allowed us to assess non-specific binding of antibodies to the plate, while the second measure revealed non-specific signal generated by HTT or plasma proteins.

For the Western blot assay, we first probed membranes with REVERT total protein stain

(**Supplementary Fig. 1i**) to confirm the appropriate transfer of all HTT proteins and to normalize for total protein content between gels. After confirming our running conditions, we tested the same murine plasma samples used for the ELISA optimization and confirmed the detection of HTT-reactive antibodies using multiple forms of HTT (**Supplementary Fig. 1j**). We were unable to measure antibodies in these samples when HTT lacking Exon1 was used as the antigen. This is an informative control as the mice were injected with Exon1 fibrils and had, therefore, never been exposed to regions outside of Exon1. Thus, the lack of antibody response supports the specificity of our assay. We then probed our Western blots with plasma from our cohort of participants and confirmed our conditions were appropriate for the detection of antibodies in human samples (**Supplementary Fig. 1k**). Finally, to confirm test-retest reliability, three separate Western blots were incubated with plasma samples from two HD patients and two controls and quantified (**Supplementary Fig. 1l**). The findings from this experiment confirmed that values were consistent between the three experiments (**Supplementary Fig. 1l**).

Together, these two complementary approaches provided a sensitive HTT-reactive antibody detection platform yielding information on antibody specificity to native (ELISA) or denatured HTT protein (Western blot assay).

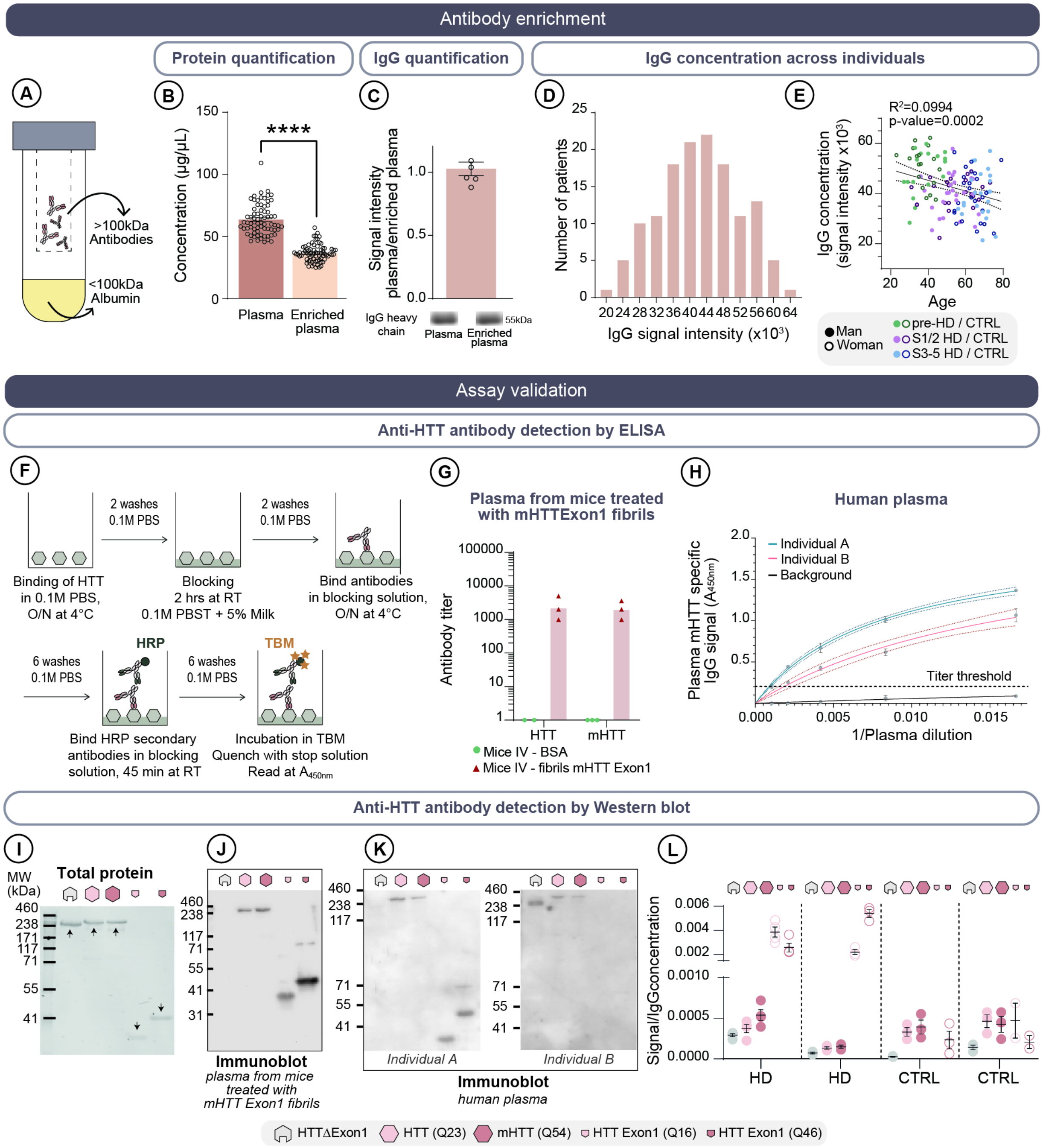

Supplementary Figure 1. Methodology validation. (**a**) Antibody enrichment was performed using an exclusion/concentration Amicon®Ultra-4 Centrifugal Filter Unit filter with a cut-off of 100kDa. (**b**) Total protein content was significantly reduced after purification (n=88; p=<0.0001). (**c**) The relative concentration of antibodies remained the same between plasma and enriched plasma, as demonstrated by signal intensity of the IgG heavy chain measured by Western blot (n=5 different individuals). (**d**) IgG concentration was variable across patients (n=88) and therefore the HTT/mHTT-reactive antibody signal was normalized to total IgG content within plasma. (**e**)

Comparison of IgG concentration across groups revealed an increased signal of IgG in young compared to aged controls (n=88; p=0.0002). (**f**) Schematic of the ELISA assay. (**g**) Validation of the ELISA was performed using plasma from mice injected with mHTTExon1 fibrils (positive control) or BSA (negative control). (**h**) The ELISA performed on human plasma demonstrated minimal unspecific signal (background curve in black, average of A_{450}=0.05), as well as a reproducibility within individuals (n=2 individuals run in duplicate). (**i-k**) Antibody specificity was tested using Western blot analysis. (**i**) Antigenic proteins were properly transferred to the membrane as shown by total protein staining (REVERT). The presence of antibodies against HTT protein was confirmed by a band at the appropriate molecular weight in both mice injected with mHTTExon1 fibrils (positive control) (**j**) and human serum of two healthy individuals (**k**). Evaluation of test-retest reliability of the Western blot assay (n=4 individual runs in triplicate) (**l**). Statistical analyses: Wilcoxon matched pairs signed rank test (**b**), One-sample t-test with theoretical median set at 1 (**c**) simple linear regression (**e**) (**p<0.01; ****p<0.0001) (**l**) Bars represent standard deviation of the mean. Abbreviations: hr: hour; HRP: horseradish peroxidase; HTT: Huntingtin protein; IgG: Immunoglobulin gamma; kDa: kilodalton; m: minute; M: mol/L; MW: molecular weight of ladder proteins; O/N: overnight; PBS: phosphate-buffered saline; pre-HD: premanifest patient; RT: room temperature; S1-2 HD: patients presenting with early disease – stage 1-2; S3-5 HD: patients presenting with advanced disease – stage 3-5; TMB: tetramethylbenzidine; WB: Western blot.

Primary antibody	Concentration	Company	Catalog Number
Rabbit anti-Huntingtin D7F7	3.5nmol/L	Cell Signaling	#5656S
Rabbit anti-Huntingtin EPR5526	3.5nmol/L	Abcam	#ab109115
Mouse anti-Ataxin3 1H9-2	1ng/mL	Biolegend	#650401

Secondary antibody	Concentration/Dilution	Company	Catalog Number
Goat anti-rabbit HRP	ELISA: 7ng/mL	Cell Signaling	#7074
Horse anti-mouse HRP	ELISA: 1/4 000<br>Western blot: 1/20 000	Cell Signaling	#7076
Goat anti-human Fc-gamma fragment HRP	ELISA: 1/4 000<br>Western blot: 1/20 000	Jackson ImmunoResearch	#109-035-098

Supplementary Table 1. Antibodies used for ELISA or western blot assays. Summary of commercial antibodies used in this study including concentration/dilution, company and catalog number.

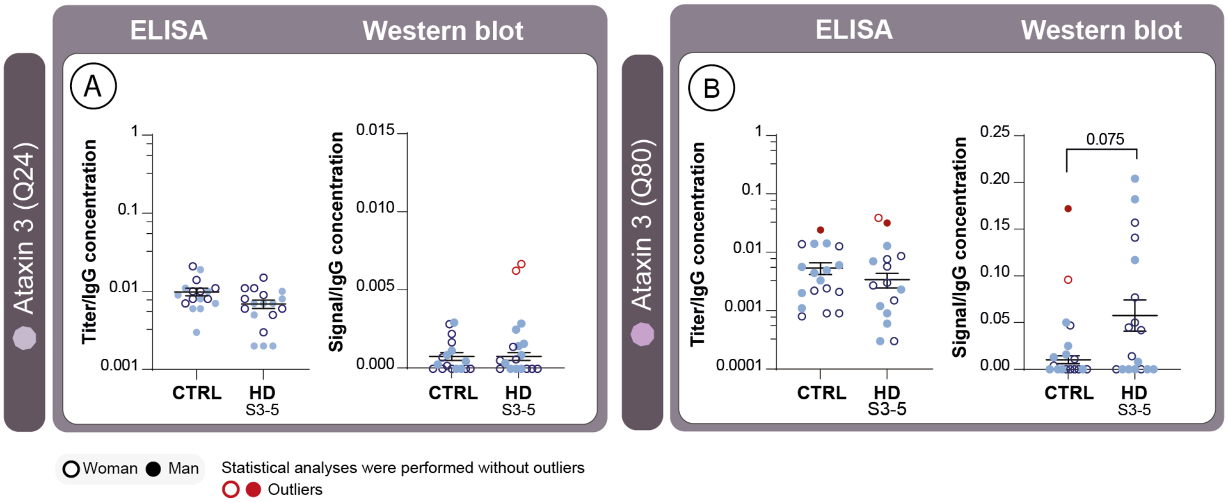

Supplementary Figure 2. Detection of antibodies against the Poly-Q containing protein Ataxin 3. Detection of antibodies against Ataxin3 containing either a normal (**A**) or expanded polyQ stretch (**B**) by ELISA or Western blot. Purified plasma from patients with advanced disease (stage 3-5, n=18: 9 women and 9 men) and their age/gender-matched healthy controls (n=18: 9 women and 9 men for each group) were tested. Statistical analyses: (**A**) Unpaired t-test with Welch's correction, (**B**) Mann-Whitney test. Abbreviations: CTRL: healthy control; IgG: Immunoglobulin gamma; S3-5 HD: patients presenting with advanced disease – stage 3-5.
